# Isofraxidin biosynthesis in *Chloranthus*: genomic insights into metabolic evolution of an early angiosperm phytoalexin

**DOI:** 10.3389/fpls.2025.1694195

**Published:** 2026-01-14

**Authors:** Yingying Liu, Lu Chen, Ping Li, Shugen Wei, Yuan Huang, Mengjin Tan, Ying Wei, Feng Peng, Yu Pan, Lisha Song, Lingyun Wan, Zhigang Yan, Lingjian Gui

**Affiliations:** 1 Guangxi Key Laboratory of High-Quality Formation and Utilization of Dao-di Herbs, Guangxi Botanical Garden of Medicinal Plants, Nanning, China; 2 National Center for Traditional Chinese Medicine (TCM) Inheritance and Innovation, Guangxi Botanical Garden of Medicinal Plants, Nanning, China; 3 National Engineering Research Center for Southwest Endangered Medicinal Materials Resources Development, Guangxi Botanical Garden of Medicinal Plants, Nanning, Guangxi, China; 4 Guangxi Botanical Garden of Medicinal Plants, Nanning, Guangxi, China

**Keywords:** *Chloranthus*, genome, metabolome-transcriptome integration, coumarin biosynthesis, isofraxidin

## Abstract

As an early-diverging angiosperm lineage, Chloranthaceae produces specialized coumarins with documented antimicrobial and anti-inflammatory activities, which contribute to its ecological success. Isofraxidin, the most representative simple coumarin in this clade, exhibits significant pharmaceutical potential. However, its biosynthetic basis remains uncharacterized. Here, we assembled a high-quality triploid genome of *Chloranthus* sp*icatus* (8.57 Gb, contig N50 = 8.76 Mb) to explore the evolution of defensive metabolism. Genomic analysis revealed an ancient whole-genome duplication event and expanded gene families associated with pathogen resistance. Metabolomic analysis identified at least 49 coumarin compounds in *Chloranthus* plants, significantly exceeding previous records. Integrated omics revealed 267 candidate biosynthetic genes across 9 enzyme families governing isofraxidin biosynthesis. Building on the upstream synthesis of the phenylpropanoid backbone, this study identifies amplified coumarin synthase (COSY) genes linked to umbelliferone accumulation, and specific CYP450s and O-methyltransferases catalyzing final structural modifications. This work elucidates the evolution of chemical defenses in early angiosperms and enables the engineering of plant-derived antimicrobials.

## 1 Introduction

The Chloranthaceae family, an early-diverging lineage of angiosperms, has long captivated biologists due to its unique combination of ancestral traits. Its vascular system exclusively contains scalariform perforation plates (Kong, 2000), a characteristic shared with ancient ANA-grade taxa of flowering plants. The frequent absence of perianth structures in Chloranthaceae flowers exhibits remarkable convergence with Piperales members (Saururaceae and Piperaceae) and basal monocots (Hughes et al., 1979; Hughes, 1994). Paleobotanical evidence positions Chloranthaceae fossils as one of the most extensively distributed early angiosperm fossil groups during the Early Cretaceous (Taylor and Hickey, 1992). The global occurrence of these fossils, particularly pollen fossils demonstrating striking morphological continuity with living Chloranthaceae species (Doyle and Endress, 2014), provides critical insights into the diversification patterns and biogeographic dispersal of the early angiosperms.

Chloranthaceae species are pharmacologically significant for their specialized metabolites, particularly diverse terpenoids (Guo et al., 2021) and coumarin derivatives (Zhang et al., 2016). Terpenoid metabolism has been relatively well-characterized in early angiosperms (Doyle and Endress, 2014; Chen et al., 2020). However, coumarin biosynthesis remains incompletely understood and persistently overlooked. The phytochemicals not only define the family’s distinctive biological properties but also play crucial roles in plant defense mechanisms. Under environmental stressors including pathogen attack, insect herbivory, nutrient deprivation, and growth restriction, Chloranthaceae species exhibit upregulated biosynthesis and compartmentalization of coumarins as an evolutionary conserved protective strategy (Robe et al., 2021). Among these secondary metabolites, isofraxidin (7-hydroxy-6,8-dimethoxycoumarin) stands out as a representative simple coumarin (Sharifi-Rad et al., 2021). As a bioactive constituent, isofraxidin demonstrates pleiotropic pharmacological activities through modulation of key inflammatory mediators: nuclear factor kappa-light-chain-enhancer of activated B cells (NF-κB), tumor necrosis factor-alpha (TNF-α), and matrix metalloproteinases (MMPs), highlighting its therapeutic potential in inflammatory regulation (Durmaz et al., 2023; He et al., 2024).

Although isofraxidin plays a crucial role in plant stress resistance and bioactivity, its biosynthetic pathway remains unresolved. While the core coumarin backbone formation is well-established in plants, the downstream pathway specific to isofraxidin faces significant challenges. This pathway requires regiospecific hydroxylation by cytochrome P450s (CYP71 family) followed by methoxylation through O-methyltransferases (OMTs). These enzyme families contain hundreds of functionally divergent members in plant genomes, making it difficult to pinpoint the exact isoforms responsible for isofraxidin’s unique 6,8-dimethoxy substitution pattern. Conventional botanical extraction remains the primary method for obtaining isofraxidin to date. However, this approach suffers from low efficiency due to the compound’s natural scarcity in plants and raises environmental sustainability concerns. Here, we generated a high-quality genome assembly of the autotriploid cultivar *Chloranthus spicatus* using multiple advanced technologies. Through comparative genomics analysis, we validated the evolutionary position of Chloranthaceae as a critical lineage in angiosperm evolution. By integrating genomics, transcriptomics, and metabolomics datasets, we elucidated the biosynthetic pathway and accumulation patterns of isofraxidin in *C.* sp*icatus*. This study establishes the genomic foundations of chemical defense evolution in early-diverging angiosperm lineages, deciphering specialized metabolic systems to advance engineered production of plant-derived antimicrobials.

## 2 Results

### 2.1 Chromosome-scale genome assembly and annotation

Using PacBio HiFi sequencing (122.62 Gb) combined with Illumina short-read data (227.40 Gb), we generated a 8.57 Gb triploid genome with 99% sequence anchored to 45 chromosomal pseudomolecules through Hi-C scaffolding (**Table 1**, **Supplementary Tables S1-S6**).

**Table 1:** Statistics of the assembly and annotation of *Chloranthus* genome.

Genome assembly	No. of sequences	Total length (bp)	N50 (bp)	N90 (bp)	Longest (bp)
Contigs	8,503	8,660,104,190	8,762,697	1,628,957	67,315,735
Hi-C assembly	1,784	8,569,334,221	178,915,312	136,432,957	336,677,673
Unplaced	1,739	85,436,445			
Chromosomes	45	8,483,897,776	178,915,312	136,432,957	336,677,673

**Figure 1:**
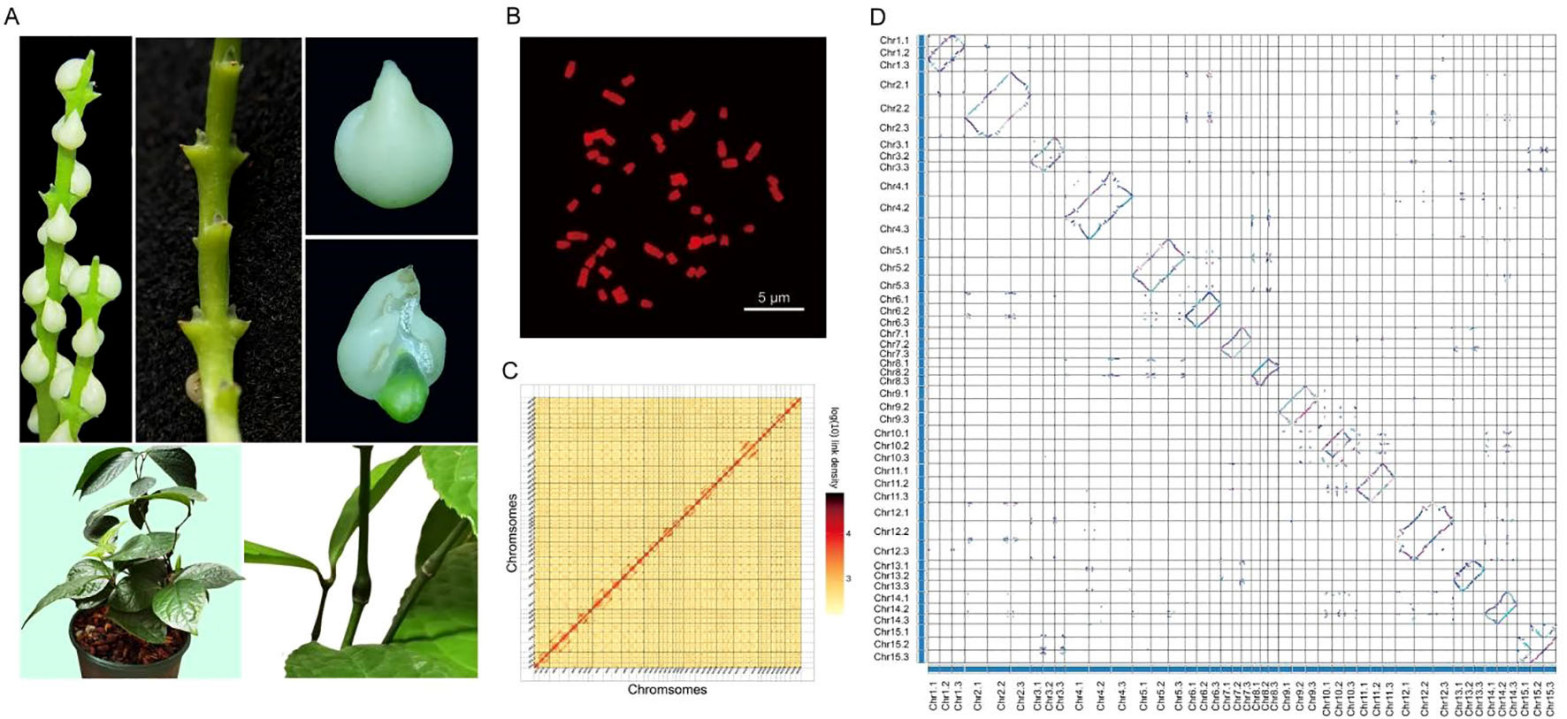
Genome assembly of *Chloranthus* and the genomic characteristics. **(A)** Morphological characteristics of flowers, leaves and stems. **(B)** The species has 45 chromosomes and the karyotype is 3x = 45. **(C)** The Hi-C heatmap of assembled chromosomes. **(D)** Syntenic blocks of homologous Chromosomes.

Chromosomal organization was validated by cytogenetic analysis (**Figure 1**, **Supplementary Figure S1**) and corroborated through *K*-mer analysis of sequencing data (**Supplementary Figure S2**, **Supplementary Table S4**), collectively confirming the triploid karyotype of 3x = 45. The assembly achieved 8.76 Mb contig N50 and 94.35% BUSCO (Manni et al., 2021) completeness (**Supplementary Tables S7, S8**), showing superior contiguity compared to other triploid plant genomes like cultivated bananas (Li et al., 2024). Integrated annotation combining transcriptomic and homology evidence identified 72,675 protein-coding genes (average CDS length 1,154 bp) with 92.7% functional annotation rate (**Supplementary Tables S9-S11**, **Supplementary Figures S3, S4**). Comparative genomic analysis indicated that both the assembly and annotation quality of the *Chloranthus* genome are robust relative to related species (**Supplementary Table S12**). Furthermore, Comparative analysis revealed high gene content conservation across homologous chromosomes, while Hi-C interaction maps resolved three-dimensional chromatin architecture.

### 2.2 Transposable element accumulation and whole genome duplication

Analysis of transposable elements (TEs) and whole-genome duplication (WGD) events revealed significant genomic evolutionary drivers in *C.* sp*icatus* (Wendel et al., 2016). Combined homolog-based and structure-based analyses identified 6315.87 Mb TEs occupying 73.7% of the assembled genome (**Supplementary Table S14**, **Supplementary Figure S5**), exceeding TE content in most angiosperms, as well as ginkgo (>70%) (Liu et al., 2021) and pine (69.4%) (Niu et al., 2022). Long terminal repeats (LTRs) dominate (63.54% of genome), suggesting slow TE clearance mechanisms similar to pine (Liu et al., 2021), contributing to the large genome size.

**Figure 2:**
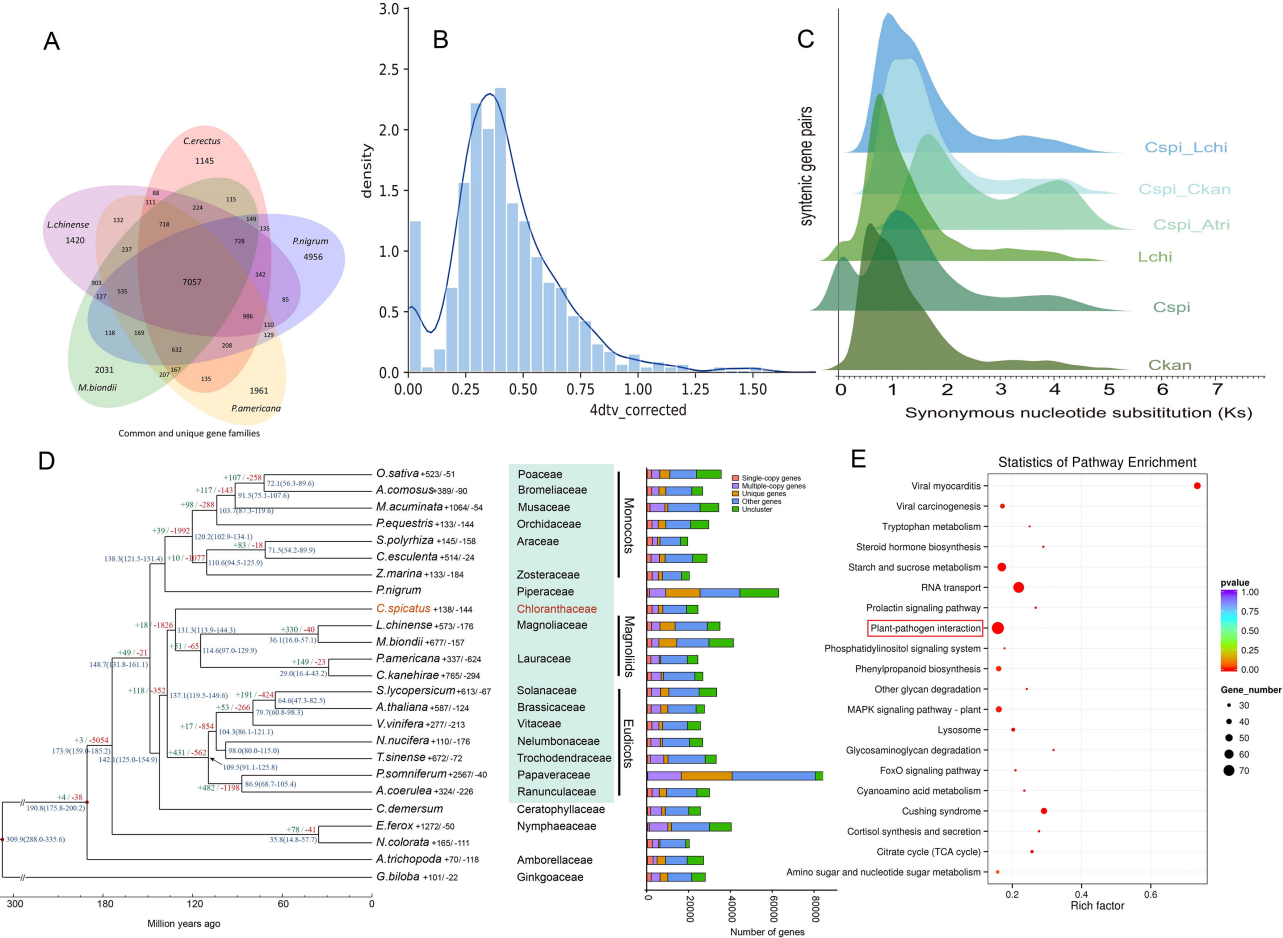
Genomic insights into the evolution of *Chloranthus*. **(A)** Shared and unique gene families between *Chloranthus* and magnoliids. **(B)** 4DTv distribution of homologous blocks. **(C)** Ks peaks reveal the specific WGD. **(D)** Phylogenomic tree of 25 representative species showing *Chloranthus* and magnoliids as sister to eudicots (±: expanded/contracted gene families). **(E)** KEGG enrichment highlights plant-pathogen interaction pathways.

Comparative genomic analysis using monoploid chromosome representatives detected a single WGD event through 4DTv and Ks distribution analyses (**Figures 2B, C**), consistent with previous findings in the diploid *Chloranthus* (Guo et al., 2021). The Ks peak at 1.1~ and calculated divergence rate (4.339821e-09/year) dated this event to 126.7 Mya. Phylogenetic comparisons with *Amborella* (Albert et al., 2013) and Magnoliaceae confirmed this paleopolyploidy event was unique to Chloranthaceae (**Figure 2D**).

### 2.3 Phylogenetic reconstruction

The phylogenetic relationships among Magnoliids, Monocots, and Eudicots continue to present unresolved questions in angiosperm evolution (Hu et al., 2019). Leveraging genomic data from early-diverging angiosperms, our study provides enhanced resolution of these critical evolutionary connections. Our comprehensive sampling encompassed 25 representative species across major plant lineages (**Supplementary Table S15**). A phylogenetically informative set of 1,092 conserved low-copy nuclear genes (LCGs) was rigorously curated from whole-genome alignments to reconstruct maximum likelihood phylogenies with robust statistical support.

*Chloranthus* demonstrated strong phylogenetic affinity with core Magnoliids, forming a well-supported group (BS = 100) that resolves as sister to the Eudicot clade (**Figure 2D**). This topology aligns with current models positioning Magnoliids as a paraphyletic lineage ancestral to core eudicots (Chaw et al., 2019). Systematic subsampling further revealed exceptional topological concordance across analytical frameworks, evidenced by consistent results from 1,092 LCGs and 517 LCGs optimized for site-heterogeneous models (**Supplementary Figure S6**). Finally, a coalescent-based species tree reconstructed from 1,092 LCGs delineated three main angiosperm lineages with high confidence: Monocots, *Chloranthus* + Magnoliids, and Eudicots.

### 2.4 Expansion of disease resistance-related gene families

The analysis of gene families showed that 48,843 gene families were clustered in 25 species, of which 3,361 gene families were shared. The corresponding clustering results of the genomes of *C.* sp*icatus* and four Magnoliids species, *P. nigrum*, *L. chinense*, *M. biondii* and *P. americana* were extracted, and it was found that the number of gene families they shared was 7,057 (**Figure 2A**), which may represent the core gene families of Chloranthales and related Magnoliids.

Comparative genomic analysis revealed the dynamic evolution of gene families in *C.* sp*icatus*, identifying 138 expanded families (encompassing 1,310 genes), including the coumarin synthase (COSY) family closely associated with coumarin biosynthesis, along with 144 contracted families (**Figure 2D**). Notably, genes related to plant-pathogen interactions were found to be significantly expanded and enriched (**Figure 2E**). The KEGG plant-pathogen interaction pathway integrates a multi-level gene network ranging from pathogen recognition (PRRs), signal transduction (MAPK, calcium signaling), transcriptional regulation (WRKY, NPR) to defense execution (ROS, PR proteins). The coordinated action of these genes helps plants balance defense and growth and resist pathogen invasion through PTI and ETI mechanisms. The coordinated expansion of these immune-related loci suggests an evolutionary arms race between *C.* sp*icatus* and its ancestral pathogens, which may explain the successful adaptation of the *Chloranthus* genus to a wide range of ecological environments.

### 2.5 Biosynthesis of isofraxidin

#### 2.5.1 Plant coumarin biosynthetic pathways and general framework

**Figure 3:**
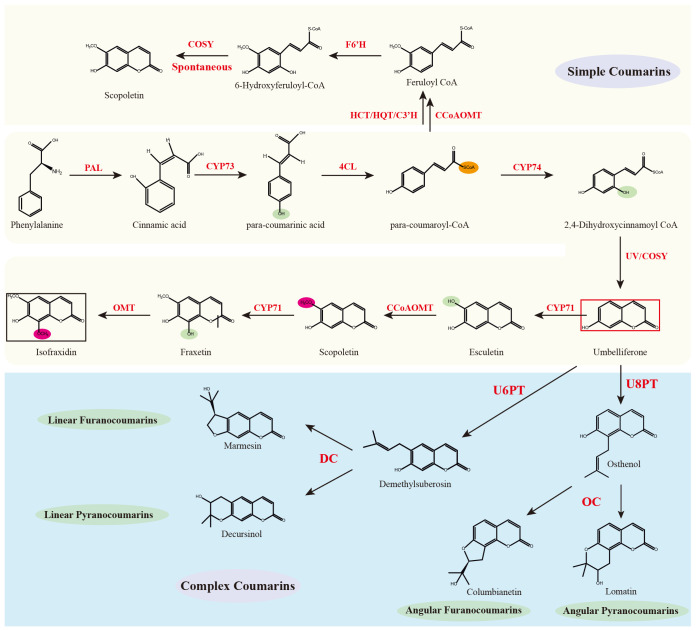
Core enzymatic framework for coumarin biosynthesis in angiosperms and the experimentally resolved pathway of isofraxidin. Red box: Universal coumarin scaffold; Black box: Isofraxidin.

The coumarin backbone is derived from phenylalanine, which undergoes deamination catalyzed by phenylalanine ammonia-lyase (PAL), resulting in the formation of trans-cinnamic acid. This intermediate is subsequently hydroxylated at the para position by cinnamic acid 4-hydroxylase (C4H, CYP73) to yield p-coumaric acid. The carboxylic acid is then activated by 4-coumaroyl-CoA ligase (4CL) to generate 4-coumaroyl-CoA (para-coumaroyl-CoA). Ortho-hydroxylation of 4-coumaroyl-CoA at the C2′ position is catalyzed by coumaroyl-CoA 2′-hydroxylase (C2′H, CYP74) to yield the unstable intermediate 2′,4′-dihydroxycinnamoyl-CoA. Recent studies have demonstrated that coumarin synthase (COSY), a member of the BAHD acyltransferase family, facilitates the spontaneous cyclization of this intermediate into umbelliferone (Wang et al., 2023), which serves as the universal scaffold for coumarin derivatives (**Figure 3**).

From umbelliferone, coumarin biosynthesis diverges into simple coumarins and complex coumarins (pyranocoumarins and furanocoumarins). Simple coumarins are mainly subjected to substitutions at positions C3–C8 and functional group modifications on the core nucleus. In contrast, the biosynthesis of complex coumarins initiates with the prenylation of umbelliferone. Prenyltransferases mediate the attachment of prenyl groups at either the C6 or C8 position, producing 6-prenylumbelliferone or 8-prenylumbelliferone, respectively (Huang et al., 2024). The 6-substituted derivatives are subsequently cyclized by angular-type cyclases to form pyranocoumarins, whereas the 8-substituted derivatives undergo cyclization by linear-type cyclases to yield furanocoumarins.

#### 2.5.2 Alignment and discrepancies between *Chloranthus* metabolomics and known pathways

Metabolome-wide profiling in *Chloranthus* identified 49 distinct coumarin metabolites (**Supplementary Table S20**), representing a quantitatively significant expansion over previously documented occurrences (Liu et al., 2022). Structurally, the majority constituted most of them are simple coumarins, such as daphnetin, fraxidin and scopolin. Additionally, we identified structurally diversified derivatives, including cleomiscosin A/C. Crucially, pyranocoumarin and furanocoumarin subclasses, which characteristic of Apiaceae and Rutaceae, were nearly absent across all parts in this species. This chemotaxonomic gap implies substantially reduced biosynthetic capability for prenylation and dehydrative cyclization reactions catalyzed by PTs and DC/OC enzymes, respectively. We propose that limited transcriptional activation or catalytically constrained orthologs of these pathway-specific enzymes result in negligible metabolic flux toward downstream heterocyclic coumarin biosynthesis.

#### 2.5.3 Integrated transcriptomic-metabolomic elucidation of the isofraxidin biosynthetic pathway

**Figure 4:**
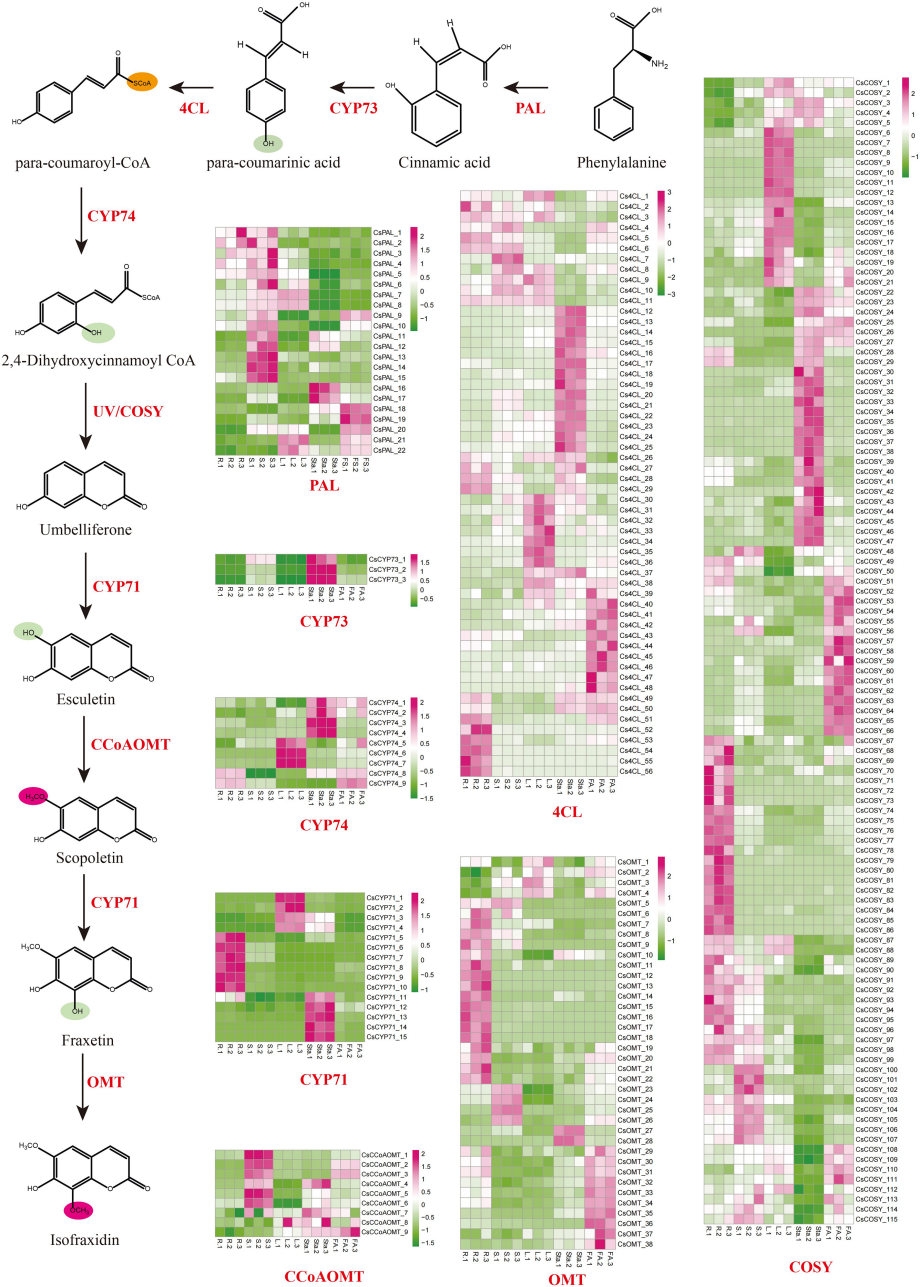
RNA-seq analysis of gene expression profiles in the *Chloranthus* isofraxidin biosynthetic pathway. Tissue-specific expression patterns of pathway genes in root (R), stem (S), leaf (L), stamen (Sta), and floral axis (FA).

To elucidate the uncharacterized biosynthetic pathway of isofraxidin, a key simple coumarin in *Chloranthus*, we integrated transcriptomic and metabolomic approaches. Through gene mining focused on umbelliferone formation and downstream modifications, we identified 267 candidates from 9 core enzyme families (PAL, CYP73, 4CL, CYP74, COSY, CYP71, CCoAOMT, OMT). Notably, the COSY family exhibited significant expansion (115 members compared to 29 in *Arabidopsis* and 44 in *Amborella*, P<0.05), while other families displayed distinct evolutionary patterns: PAL (22), CYP73 (3), C4L (56), CYP74 (9), CYP71 (15), CCoAOMT (9), and OMT (38), indicating differential gene duplication strategies among these families to meet metabolic demands during evolution. Spatial expression profiling revealed tissue-specific patterns that COSY members showed distinct expression in roots, stems, leaves, and stamens, CYP71 subgroups demonstrated root (CYP71_5-10), leaf (CYP71_1-4), and stamens (CYP71_11-15) specificity, while OMT_5–22 and OMT_23–26 exhibited predominant expression in roots and stems, respectively (**Figure 4**).

**Figure 5:**
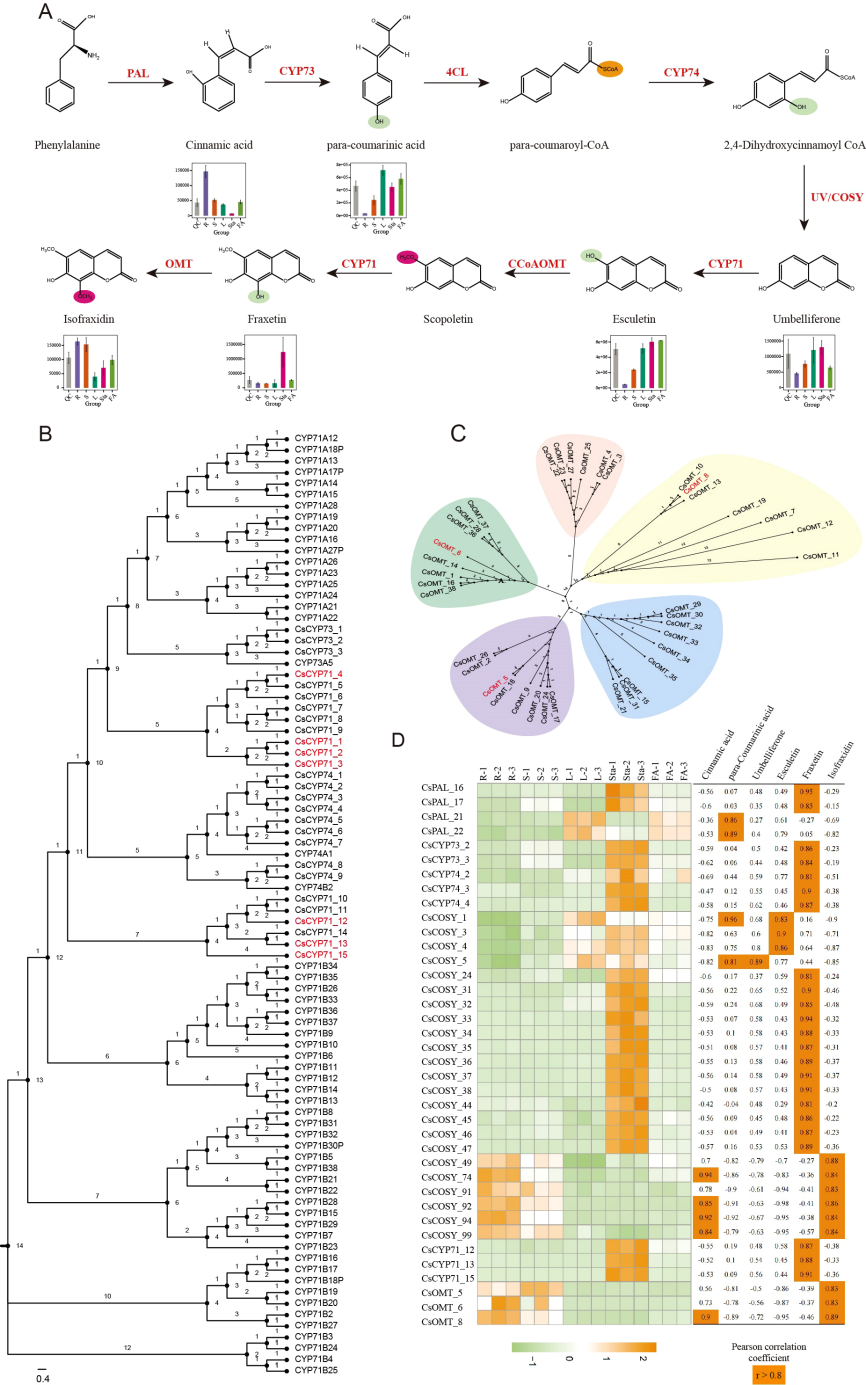
Integrative metabolomic and transcriptomic analysis. **(A)** Relative abundance of key metabolites in the isofraxidin biosynthetic pathway across different tissues: root (R), stem (S), leaf (L), stamen (Sta), and floral axis (FA). Pooled quality control (QC) samples are included. **(B, C)** Phylogenetic trees of candidate P450s and OMTs. Key enzyme genes are highlighted in red. **(D)** Expression patterns of isofraxidin synthesis-associated genes. Strong positive correlations (Pearson *r* ≥ 0.8, *p* < 0.05, *n* = 3) are indicated by orange backgrounds.

UPLC-MS/MS analysis validated the spatial accumulation of key pathway intermediates. PAL-mediated conversion of phenylalanine to cinnamic acid exhibited peak catalytic activity in root tissues, correlating with significant substrate accumulation. Downstream CYP73 hydroxylation generated para-coumarinic acid, which preferentially localized to leaves, stamens, and floral axes. Subsequent CYP74 catalysis yielded 2,4-dihydroxycinnamic acid, followed by COSY-driven cyclization to umbelliferone. Metabolomic profiling revealed umbelliferone levels were 2-fold higher in leaves and stamens versus roots, with biosynthesis primarily driven by COSY_1–21 and COSY_22–47 clusters (**Figure 5A**). Integrated transcriptome-metabolome analysis demonstrated strong positive correlations between COSY expression and umbelliferone concentrations (r > 0.85), confirming their essential role in scaffold formation.

Critical downstream steps were resolved through multi-omics integration. CYP71_1-4 (leaf-specific, FPKM>1) catalyzed umbelliferone hydroxylation to esculetin. Strikingly, stamen-specific CYP71_11-15 (FPKM>1) mediated the conversion of esculetin-derived intermediates to fraxetin, with CYP71_12/13/15 expression strongly correlating with fraxetin accumulation (*r* = 0.87–0.91; **Figures 5B, D**). Concurrent suppression of CCoAOMT in stamens (FPKM<1) diverted metabolic flux from lignin precursors toward isofraxidin biosynthesis. The terminal methylation of fraxetin to isofraxidin was attributed to root/stem-enriched OMT_5/6/8 (FPKM>1), whose expression tightly correlated with product accumulation (*r* = 0.83 – 0.89; **Figures 5C, D**).

Collectively, these results demonstrate a compartmentalized biosynthetic pathway wherein hydroxylation occurs primarily in photosynthetic (leaves) and reproductive (stamens) tissues, while methylation is mainly limited to structural organs (roots/stems). This spatial segregation minimizes metabolic crosstalk while optimizing resource allocation.

## 3 Discussion

Herbal genomics, an emerging research field, investigates the genetic and regulatory mechanisms of medicinal plants through genomic approaches to elucidate their bioactive principles and advance molecular breeding (Leng et al., 2024; Ouadi et al., 2022). Genomic dissection of valuable natural product biosynthetic pathways provides critical insights for synthetic biology-driven compound synthesis and scalable production. Co-expression network analysis and genome mining are becoming indispensable strategies to accelerate the modernization of traditional medicinal plant research.

The biosynthesis of coumarins and associated genes has evolved independently multiple times in plants (Huang et al., 2024). As an early-diverging angiosperm, *Chloranthus* accumulates diverse simple coumarins, among which isofraxidin—a compound with extensive clinical applications and significant pharmaceutical potential—warrants systematic investigation. Through integrated multi-omics analysis, this study elucidates the genetic basis of isofraxidin biosynthesis, offering the first comprehensive understanding of its metabolic regulation. Our findings reveal the remarkable complexity and evolutionary adaptability of plant secondary metabolism in *Chloranthus*. Systematic identification of 9 key gene families (267 candidate genes) and their functional specialization within the metabolic cascade provides novel perspectives on coumarin regulation.

Recent studies have established the COSY-encoded enzyme as catalytically essential for coumarin biosynthesis in upstream pathway steps, revising the conventional model wherein cyclization was considered spontaneous (Wang et al., 2023). Consequently, COSY gene copy number expansion likely enhances umbelliferone production capacity. Notably, coumarin abundance exhibits significant divergence across angiosperm lineages, and the amplification of the COSY gene family, providing the core scaffold for bioactive coumarins, constitutes a pivotal driver of this metabolic diversification.

This amplification is strikingly exemplified in *Chloranthus*. While *Arabidopsis thaliana* possesses 29 and *Amborella trichopoda* 44 COSY orthologs, the *Chloranthus* genome exhibits substantial expansion with 115 members—highlighting a dramatic lineage-specific proliferation. Although the *Chloranthus* lineage has undergone a clade-specific WGD event, the expansion of COSY is more likely attributable to tandem duplications or other local replication mechanisms rather than WGD. This significant disparity in gene family size underscores key expansion events during plant evolution, driving functional diversification. Such genomic alterations provide the raw material for metabolic innovation, enabling novel traits like enhanced biotic stress countermeasures (such as pathogen defense), while environmental pressures act as selective filters fixing advantageous variants.

Our analysis of the CYP71 subfamily reveals tissue-specific functional partitioning among its members. Subclades CYP71_1–4 exhibit high expression in leaf tissues, where they catalyze the hydroxylation of umbelliferone to yield esculetin. Conversely, isoforms CYP71_11–15 demonstrate stamen-specific expression and drive the conversion of scopoletin to fraxetin. This metabolic modularity strategy effectively minimizes cytotoxicity risks by confining potentially toxic intermediates (such as esculetin) to specialized tissues, while optimizing metabolic flux through spatial compartmentalization. Consequently, defense compound biosynthesis achieves precise spatiotemporal regulation.

Gene family functional stratification is equally notable. The final step of isofraxidin biosynthesis requires an OMT for methylation. Transcriptomics identified OMT_5, OMT_6, and OMT_8 with rhizome-specific high expression (FPKM > 1), showing strong positive correlation with isofraxidin accumulation (*r* = 0.83–0.89, P < 0.05). Among 38 screened OMT genes, only these three core members significantly associate with target metabolite production. This finding indicates strict spatiotemporal and functional stratification within the OMT family. Core isoforms OMT_5/6/8 specifically dominate isofraxidin biosynthesis in rhizomes, while paralogs participate in divergent pathways—such as lignin synthesis (*Eucalyptus* CCoAOMT homologs) or flavonoid modification (*Citrus* CrcCCoAOMT7 homologs) (Carocha et al., 2015; Peng et al., 2024).

Collectively, this study elucidates the biosynthetic pathway of isofraxin, a key coumarin in *Chloranthus*, and substantiates the paradigm of “one gene family, multiple functions; one metabolic pathway, multiple genes.” This genomic plasticity-driven mechanism of metabolic innovation likely represents a pivotal evolutionary strategy that facilitated the ecological success of early angiosperms, including members of the Chloranthaceae family, in response to the complex environmental pressures of the Cretaceous. We further highlight key genes in the coumarin biosynthetic pathway that display lineage-specific expansion or elevated expression in relevant tissues as prime candidates for functional characterization. Future studies should employ CRISPR/Cas9-mediated knockout, RNAi silencing, or overexpression techniques in plant models or heterologous systems (such as *Escherichia coli* or yeast) to functionally validate COSY genes, members of the CYP71 subfamily, and core OMT genes. These efforts will help clarify their precise catalytic functions and regulatory roles within coumarin biosynthesis.

## 4 Materials and methods

### 4.1 Materials and sequencing

Fresh leaves were collected from one individual of *C.* sp*icatus* (LYY202008). The samples were sent to Novogene (Beijing, China) for DNA extraction and sequencing. Chromosomes were checked using root tips from plants. After staining with DAPI, photographs were taken under a fluorescent microscope (Leica DM2500) in dark. Determine its karyotype as 3X = 45. Genome size and heterozygosity was estimated using *K*-mer analysis of Illumina 150 bp paired-end reads. The *K*-mer depth-frequency distribution was generated using jellyfish v.2.2.7 (Marçais and Kingsford, 2011).

DNA was extracted from leaves using the DNAsecure Plant Kit (TIANGEN). The 15 Kb circular consensus sequencing (CCS) library was constructed and sequenced on the PacBio Sequge II platform. Short reads genomic library was prepared and sequenced using the Illumina HiSeq platform. Young leaf samples were processed and DNA extracted using standard protocols, and a 350 bp Hi-C library was sequenced on an Illumina HiSeq instrument.

Roots (R), stems (S), leaves (L), stamens (Sta), and floral axis (FA) under normal growth conditions were collected for metabolomics detection and transcriptome sequencing.

### 4.2 Genome assembly

The 122.62 Gb (7 cells) Hifi reads were rapidly constructed using hifiasm v.0.14 (Cheng et al., 2021). In order to evaluate the accuracy of the assembly, the reads of the small fragment library were aligned to the assembled genome using BWA v.0.7.10 (Li and Durbin, 2009), and the alignment rate, the coverage of genome and the distribution of depth were counted. The presence of contamination was assessed using GC content and sequencing coverage analysis. We applied both CEGMA v.2.5 (Parra et al., 2007) and BUSCO v.3.0 (Simão et al., 2015) to assess the integrity of the assembly.

Hi-C data (510 Gb) was obtained on the Illumina HiSeq platform, and ALLHIC (Zhang et al., 2019) was used for contig clustering, ranking and orientation. The preliminary scaffolding was primarily performed using 3D-DNA and Juicer for automated processing. Following the initial chromosome-scale assembly, we rigorously evaluated the quality of each chromosome using the Hi-C contact matrix. Misassemblies or misorientations were identified based on the following criteria: a sharp discontinuity or significant deviation in the intensity of intra-chromosomal interactions, and discontinuous or anomalous interaction patterns between adjacent scaffolds. Manual corrections were applied to the suspected regions in Juicebox v.1.11.08 (Durand et al., 2016) according to the strength of chromosome interactions, and the effectiveness of each correction was subsequently verified by re-examining the Hi-C heatmap. This iterative process ensured the reproducibility of the correction methodology. Only minor adjustments were required for a limited number of anomalous regions. The final triploid chromosome assembly was generated, containing all 45 chromosomes.

### 4.3 Repeat annotation

We used both homology-based and *de novo*-based strategies to identify transposable elements (TEs). Firstly, RepeatMasker v.4.0.7 (Chen, 2004) and RepeatProteinMask are used to generate homology-based repeat libraries based on RepBase nucleic acid library and RepBase protein library, respectively. *De novo* predictions are then performed using RepeatModeler v.1.0.5 (Flynn et al., 2020), RepeatScout (Price et al., 2005), Piler (Edgar and Myers, 2005) and LTR_FINDER v.1.0.6 (Xu and Wang, 2007). All TEs data were integrated and de-redundant to obtain an integrated repeat library, which was finally annotated by RepeatMasker.

### 4.4 Protein-coding gene prediction and functional annotation

Three complementary strategies, including denovo, homology, and RNA-seq based prediction were used to annotate the protein-coding genes of the *C.* sp*icatus* genome. Augustus v.3.0.2 (Stanke et al., 2006), Genscan v.1.0 (Li et al., 2010), Geneid (Parra et al., 2000), GlimmerHMM v.3.0.3 (Majoros et al., 2004) and SNAP (Korf, 2004) were run on the repeat-masked genomes to evaluate *de novo* gene predictions. For homolog-based prediction, we used the inferred protein sequences of four species, *C. demersum*, *L. chinense*, *N. colorata* and *P. somniferum*. Alignments were further processed using GeneWise v.2.2.0 (Birney and Durbin, 2000) to generate accurate exon and intron information. For transcriptome-based prediction, cufflinks v.2.1.1 (Trapnell et al., 2010) and PASA 2.0.2 (Haas et al., 2003) were used to predict and improve the gene structures. All predictions were combined using EVidenceModeler (EVM) v.1.1.1 (Haas et al., 2008) to generate a non-redundant gene set, resulting in a final set of 72,675 protein-coding genes.

Functional annotation of protein-coding genes was performed by performing BLASTP searches in the SwissProt (http://www.uniprot.org/), Nr (http://www.ncbi.nlm.nih.gov/protein), Pfam (http://pfam.xfam.org/), KEGG (http://www.genome.jp/kegg/) and InterPro (https://www.ebi.ac.uk/interpro/) protein databases. GO (Ashburner et al., 2000) terms for genes were obtained from InterPro entries and the KEGG (Kanehisa and Goto, 2000) pathway was generated using the KEGG database.

### 4.5 Construction of gene families

We selected 25 species (A. trichopoda, A. comosus, A. coerulea, A. thaliana, C. demersum, C. kanehirae, C. esculenta, E. ferox, G. biloba, C. spicatus, L. chinense, M. biondii, M. acuminata, N. nucifera, N. colorata, O. sativa, P. somniferum, P. americana, P. equestris, P. nigrum, S. lycopersicum, S. polyrhiza, T. sinense, V. vinifera, Z. marina) to construct gene families. Only the transcript with the longest coding region was reserved, and the similarity between protein sequences was obtained by all-vs-all blastp. Gene family clusters based on 25 species were then constructed using OrthoMCL v.2.0.9 (Li et al., 2003) with an inflation factor set as 1.5. Gene family expansion and contraction analysis was performed using CAFÉ v.4.2 (Han et al., 2013).

### 4.6 Phylogenetic analyses

The SCG and LCG of 25 seed plants were identified using SonicParanoid v.1.0 (Cosentino and Iwasaki, 2019) and OrthoMCL v.2.0.9 (Li et al., 2003). Finally, we identified 1092, 517, 299 and 27 homologous genes, respectively. Amino acid sequences were aligned using MUSCLE v.3.8.31 (Edgar, 2004). For concatenated datasets, ModelFinder (Kalyaanamoorthy et al., 2017) is used to automatically select the best-fit surrogate model. Maximum likelihood trees were inferred from the sequences using RaxML v.8.2.12 (Stamatakis, 2014), and support values were estimated using 500 bootstrap replicates. In the analysis based on coalescent approach, each gene tree was first constructed using IQ-TREE v.1.6.9 (Nguyen et al., 2015), and then these trees are used to infer species tree with posterior probabilities in Astral v.5.6.1 (Mirarab et al., 2014). To estimate the timescales of the evolution of *Chloranthus*, Magnoliids, Monocots and Eudicots, we calibrated a relaxed molecular clock with 2 well-established constraints: the divergence between angiosperms and gymnosperms (337–289 Ma) and the divergence between *A. trichopoda* and *N. colorata* (199–173 Ma) (http://www.timetree.org/). Bayesian phylogenetic age analysis and approximate likelihood calculations for branch lengths were performed on selected genes using the program MCMCTree in PAML v.4.9 (Yang, 2007; Reis and Yang, 2011).

### 4.7 Identification of whole-genome duplication

We selected four genomes of *C.* sp*icatus*, *A. trichopoda*, *L. chinense* and *C. kanehirae* for polyploidy analysis based on previous studies (Chaw et al., 2019; Chen et al., 2019). For protein BLASTP within or between genomes, the cut-off value of e value is 1×10-5. According to the position of the genes and BLASTP results, McscanX v.2 (Wang et al., 2012) was used to search for the collinear segment to determine homologous gene pairs. Protein-gene pairs were subjected to multiple sequence alignment in MUSCLE v.3.8.31 (Kalyaanamoorthy et al., 2017). The KS and 4DTv values for each homologous gene pair were estimated using the codeml method implemented in PAML v.4.9 (Yang, 2007). The distributions of the values were obtained by kernel function analysis, and they were further modeled as a mixture of multiple normal distributions by the kernel smoothed density function. Multimodal fitting of the curve was performed using the Gaussian approximation function (CfTool) in MATLAB.

### 4.8 UPLC/QTRAP-MS metabolomic analysis

Lyophilized tissues (root, stem, leaf, stamen, floral axis; 50 mg/sample) were pulverized at 30 Hz for 1.5 min (MM 400 grinder, Retsch), then extracted with 1200 μL of -20°C pre-cooled 70% methanol containing internal standards. The internal standard used was 2-chlorophenylalanine (purity: 98%, supplier: J&K Scientific, Batch No.: LBCOR15, CAS: 14091-11-3) at a concentration of 1 mg/L (1 ppm). After vortexing every 30 min (6 cycles, 30 sec each), extracts were centrifuged (12,000 rpm, 3 min), filtered through 0.22-μm membranes, and stored at -80°C. Chromatographic separation used an Agilent SB-C18 column (1.8 μm, 2.1×100 mm) with mobile phase A (0.1% formic acid/water) and B (0.1% formic acid/acetonitrile) at 0.35 mL/min (40°C). The gradient program was: 0–9 min (95%→5% A), 9–10 min (5% A), 10-11.1 min (5%→95% A), 11.1–14 min (95% A). MS detection employed an ExionLC™ AD/UPLC-ESI-QTRAP system with ion spray voltage ±5500/4500 V, source temperature 550°C, gas pressures (GSI:50 psi, GSII:60 psi, CUR:25 psi), and collision-activated dissociation in high mode. Metabolites were quantified via MRM with nitrogen collision gas, optimized declustering potential (DP), and collision energy (CE).

Metabolite identification was performed by matching the accurate mass, MS/MS fragments, isotopic distribution, and retention time (RT) of the experimental spectra against a commercial metabolite database (MetWare Database) using an intelligent MS/MS spectrum matching algorithm. Mass tolerances were set at 20 ppm for precursor ions and 20 ppm for fragment ions.

For relative quantification in MRM mode, the first quadrupole (Q1) selected target precursor ions, excluding ions from other molecules to minimize interference. The selected precursor ions were then fragmented in the collision cell, and the third quadrupole (Q3) filtered a characteristic product ion for each metabolite. The signal intensity of this specific product ion was used for quantification. After data acquisition, the peak areas for all metabolites were integrated. The raw mass spectrometry data were processed using MultiQuant software (v 3.0.2), where peaks corresponding to the same metabolite across different samples were aligned and corrected. The relative content of each metabolite was subsequently expressed based on its respective peak area.

### 4.9 Identification of gene families involved in isofraxidin biosynthesis

In the identification of gene families involved in the biosynthesis of isofraxidin pathway enzymes a comprehensive approach was adopted. For genes encoding P450 enzymes including CYP71, CYP73, and CYP74 sequences from *Arabidopsis* (https://www.arabidopsis.org) were used as references for genome-wide screening followed by sequence alignment using MAFFT and phylogenetic reconstruction with IQ-TREE v.1.6.9 applying the Approximate-Maximum-Likelihood method to identify candidate sequences clustering with AtCYP71, AtCYP73, and AtCYP74. In parallel for PAL (PF00221), COSY (PF02458), CCoAOMT (PF01596), and OMT (PF00891) initial candidate sequences were identified through HMMER v3.0 (Potter et al., 2018) searches against Pfam domains with an E-value cutoff of 1e-15 and further validated using BLASTp against specific *Arabidopsis* protein sequences AAC18870.1 (PAL), AT1G28680 (COSY), AAM66108.1 (CCoAOMT) and AT5G54160 (OMT) respectively also with an E-value threshold of 1e-15. The final list of candidate genes for each family was established by intersecting results obtained from both HMMER and BLASTp searches.

### 4.10 Integrated transcriptome-metabolome analysis

The quantitative values of both genes and metabolites across all samples were normalized using the Z-score method. Pearson correlation coefficients between gene expression and metabolite levels were calculated using the core function in R. Correlations with an absolute Pearson correlation coefficient greater than 0.8 and a p-value less than 0.05 were considered significant and selected for further analysis.

## 5 Conclusions

As an early-diverging angiosperm lineage, *Chloranthus* provides an exceptional model for investigating isofraxidin biosynthesis, offering critical insights into the adaptive evolution of chemical defenses in basal flowering plants. Through integrated multi-omics analysis complemented by enzymatic verification, we have elucidated the core regulatory framework governing representative hydroxycoumarin biosynthesis in *C.* sp*icatus*. Principal mechanisms were identified: (1) Functional divergence within the expanded COSY gene family facilitates tissue-specific accumulation of key precursors through substrate specialization, establishing a dynamic metabolic reservoir for downstream isofraxidin production. (2) CYP71 subfamily members demonstrate spatiotemporal differentiation, with stamen-enriched CYP71_12/13/15 (r > 0.87) serving as critical nodes for fraxetin biosynthesis via compartmentalized expression patterns. (3) The final modifications is achieved through rhizome-preferential OMT isoforms (OMT_5/6/7, r > 0.83), enabling accumulation patterns of terminal derivatives.

This work establishes a mechanistic paradigm for coumarin pathway evolution. By bridging genomic innovation with ecological adaptation, these findings provide advances in understanding early angiosperm chemical evolution and developing biotechnological applications for natural product biosynthesis. However, experimental validation of top candidate genes exhibiting lineage-specific expansions or high expression remains a current limitation. Future studies could employ CRISPR/Cas9-mediated gene knockout or overexpression in model systems, or conduct heterologous expression in yeast or Nicotiana benthamiana, to functionally validate key genes. Such approaches would generate further empirical evidence for reconstructing the chemical evolutionary trajectory of early angiosperms.

## Data Availability

The datasets presented in this study can be found in online repositories. The names of the repository/repositories and accession number(s) can be found in the article/**Supplementary Material**.

## References

[B1] Albert V. A. Barbazuk W. B. Depamphilis C. W. Der J. P. Leebens-Mack J. Ma H. . (2013). The *Amborella* genome and the evolution of flowering plants. Science 342, 1241089. doi: 10.1126/science.1241089, PMID: 24357323

[B2] Ashburner M. Ball C. A. Blake J. A. Botstein D. Butler H. Cherry J. M. . (2000). Gene ontology: tool for the unification of biology. Nature genetics 25, 25–29. doi: 10.1038/75556, PMID: 10802651 PMC3037419

[B3] Birney E. Durbin R. (2000). Using GeneWise in the Drosophila annotation experiment. Genome Res. 10, 547–548. doi: 10.1101/gr.10.4.547, PMID: 10779496 PMC310858

[B4] Carocha V. Soler M. Hefer C. Cassan-Wang H. Fevereiro P. Myburg A. A. . (2015). Genome-wide analysis of the lignin toolbox of *Eucalyptus grandis*. New Phytol. 206, 1297–1313. doi: 10.1111/nph.13313, PMID: 25684249

[B5] Chaw S. M. Liu Y. C. Wu Y. W. Wang H. Y. Lin C. Y. I. Wu C. S. . (2019). Stout camphor tree genome fills gaps in understanding of flowering plant genome evolution. Nat. Plants 5, 63–73. doi: 10.1038/s41477-018-0337-0, PMID: 30626928 PMC6784883

[B6] Chen N. S. (2004). Using Repeat Masker to identify repetitive elements in genomic sequences. Curr. Protoc. Bioinf. 5, 4–10. doi: 10.1002/0471250953.bi0410s05, PMID: 18428725

[B7] Chen J. Hao Z. Guang X. Zhao C. Wang P. Xue L. . (2019). *Liriodendron* genome sheds light on angiosperm phylogeny and species–pair differentiation. Nat. Plants 5, 18–25. doi: 10.1038/s41477-018-0323-6, PMID: 30559417 PMC6784881

[B8] Chen Y. C. Li Z. Zhao Y. X. Gao M. Wang J. Y. Liu K. W. . (2020). The *Litsea* genome and the evolution of the laurel family. Nat. Commun. 11, 1675. doi: 10.1038/s41467-020-15493-5, PMID: 32245969 PMC7125107

[B9] Cheng H. Concepcion G. T. Feng X. W. Zhang H. W. Li H. (2021). Haplotype-resolved *de novo* assembly using phased assembly graphs with hifiasm. Nat. Methods 18, 170–175. doi: 10.1038/s41592-020-01056-5, PMID: 33526886 PMC7961889

[B10] Cosentino S. Iwasaki W. (2019). SonicParanoid: fast, accurate and easy orthology inference. Bioinformatics 35, 149–151. doi: 10.1093/bioinformatics/bty631, PMID: 30032301 PMC6298048

[B11] Doyle J. A. Endress P. K. (2014). Integrating Early Cretaceous fossils into the phylogeny of living angiosperms: ANITA lines and relatives of Chloranthaceae. Int. J. Plant Sci. 175, 555–600. doi: 10.1086/675935

[B12] Durand N. C. Shamim M. S. Machol I. Rao S. S. Huntley M. H. Lander E. S. . (2016). Juicer provides a one-click system for analyzing loop-resolution Hi-C experiments. Cell Syst. 3, 95–98. doi: 10.1016/j.cels.2016.07.002, PMID: 27467249 PMC5846465

[B13] Durmaz L. Gulçin İ. Taslimi P. Tüzün B. (2023). Isofraxidin: antioxidant, anti-carbonic anhydrase, anti-cholinesterase, anti-diabetic, and in silico properties. ChemistrySelect 8, e202300170. doi: 10.1002/slct.202300170

[B14] Edgar R. C. (2004). MUSCLE: multiple sequence alignment with high accuracy and high throughput. Nucleic Acids Res. 32, 1792–1797. doi: 10.1093/nar/gkh340, PMID: 15034147 PMC390337

[B15] Edgar R. C. Myers E. W. (2005). PILER: identification and classification of genomic repeats. Bioinformatics 21, i152–i158. doi: 10.1093/bioinformatics/bti1003, PMID: 15961452

[B16] Flynn J. M. Hubley R. Goubert C. Rosen J. Clark A. G. Feschotte C. . (2020). RepeatModeler2 for automated genomic discovery of transposable element families. Proc. Natl. Acad. Sci. 117, 9451–9457. doi: 10.1073/pnas.1921046117, PMID: 32300014 PMC7196820

[B17] Guo X. Fang D. Sahu S. K. Yang S. Guang X. Folk R. . (2021). *Chloranthus* genome provides insights into the early diversification of angiosperms. Nat. Commun. 12, 6930. doi: 10.1038/s41467-021-26922-4, PMID: 34836973 PMC8626473

[B18] Haas B. J. Delcher A. L. Mount S. M. Wortman J. R. Smith R. K. Hannick L. I. . (2003). Improving the *Arabidopsis* genome annotation using maximal transcript alignment assemblies. Nucleic Acids Res. 31, 5654–5666. doi: 10.1093/nar/gkg770, PMID: 14500829 PMC206470

[B19] Haas B. J. Salzberg S. L. Zhu W. Pertea M. Allen J. E. Orvis J. . (2008). Automated eukaryotic gene structure annotation using EVidenceModeler and the Program to Assemble Spliced Alignments. Genome Biol. 9, R7. doi: 10.1186/gb-2008-9-1-r7, PMID: 18190707 PMC2395244

[B20] Han M. V. Thomas G. W. Lugo-Martinez J. Hahn M. W. (2013). Estimating gene gain and loss rates in the presence of error in genome assembly and annotation using CAFE 3. Mol. Biol. Evol. 30, 1987–1997. doi: 10.1093/molbev/mst100, PMID: 23709260

[B21] He S. Zhang T. Wang Y. Yuan W. Li L. Li J. . (2024). Isofraxidin attenuates dextran sulfate sodium-induced ulcerative colitis through inhibiting pyroptosis by upregulating Nrf2 and reducing reactive oxidative species. Int. Immunopharmacol. 128, 111570. doi: 10.1016/j.intimp.2024.111570, PMID: 38280336

[B22] Hu L. Xu Z. Wang M. Fan R. Yuan D. Wu B. . (2019). The chromosome-scale reference genome of black pepper provides insight into piperine biosynthesis. Nat. Commun. 10, 4702. doi: 10.1038/s41467-019-12607-6, PMID: 31619678 PMC6795880

[B23] Huang X. Tang H. Wei X. He Y. Hu S. Wu J. . (2024). The gradual establishment of complex coumarin biosynthetic pathway in Apiaceae. Nat. Commun. 15, 6864. doi: 10.1038/s41467-024-51285-x, PMID: 39127760 PMC11316762

[B24] Hughes N. F. (1994). The enigma of angiosperm origins Vol. 1 (Cambridge, UK: Cambridge University Press).

[B25] Hughes N. F. Ge D. Laing J. F. (1979). Barremian earliest angiosperm pollen. Palaeontology 22, 513–535.

[B26] Kalyaanamoorthy S. Minh B. Q. Wong T. K. F. Von Haeseler A. Jermiin L. S. (2017). ModelFinder: fast model selection for accurate phylogenetic estimates. Nat. Methods 14, 587–589. doi: 10.1038/nmeth.4285, PMID: 28481363 PMC5453245

[B27] Kanehisa M. Goto S. (2000). KEGG: kyoto encyclopedia of genes and genomes. Nucleic acids research. 28, 27–30. doi: 10.1093/nar/28.1.27, PMID: 10592173 PMC102409

[B28] Kong H. Z. (2000). Karyotypes of sarcandra gardn. and *chloranthus* swartz (Chloranthaceae) from China. Botanic J. Linn. Soc. 133, 327–342. doi: 10.1111/j.1095-8339.2000.tb01549.x

[B29] Korf I. (2004). Gene finding in novel genomes. BMC Bioinf. 5, 59. doi: 10.1186/1471-2105-5-59, PMID: 15144565 PMC421630

[B30] Leng L. Xu Z. Hong B. Zhao B. Tian Y. Wang C. . (2024). Cepharanthine analogs mining and genomes of *Stephania* accelerate anti-coronavirus drug discovery. Nat. Commun. 15, 1537. doi: 10.1038/s41467-024-45690-5, PMID: 38378731 PMC10879537

[B31] Li H. Durbin R. (2009). Fast and accurate short read alignment with Burrows-Wheeler transform. Bioinformatics 25, 1754–1760. doi: 10.1093/bioinformatics/btp324, PMID: 19451168 PMC2705234

[B32] Li L. Stoeckert C. J. Roos D. S. (2003). OrthoMCL: identification of ortholog groups for eukaryotic genomes. Genome Res. 13, 2178–2189. doi: 10.1101/gr.1224503, PMID: 12952885 PMC403725

[B33] Li X. Yu S. Cheng Z. Chang X. Yun Y. Jiang M. . (2024). Origin and evolution of the triploid cultivated banana genome. Nat. Genet. 56, 136–142. doi: 10.1038/s41588-023-01589-3, PMID: 38082204

[B34] Li R. Zhu H. Ruan J. Qian W. Fang X. Shi Z. . (2010). *De novo* assembly of human genomes with massively parallel short read sequencing. Genome Res. 20, 265–272. doi: 10.1101/gr.097261.109, PMID: 20019144 PMC2813482

[B35] Liu Y. Li Y. Huang S. Zhang H. Deng C. Song X. . (2022). Genus *Chloranthus*: A comprehensive review of its phytochemistry, pharmacology, and uses. Arabian J. Chem. 15, 104260. doi: 10.1016/j.arabjc.2022.104260

[B36] Liu H. Wang X. Wang G. Cui P. Wu S. Ai C. . (2021). The nearly complete genome of Ginkgo biloba illuminates gymnosperm evolution. Nat. Plants 7, 748–756. doi: 10.1038/s41477-021-00933-x, PMID: 34135482

[B37] Majoros W. H. Pertea M. Salzberg S. L. (2004). TigrScan and GlimmerHMM: two open source ab initio eukaryotic gene-finders. Bioinformatics 20, 2878–2879. doi: 10.1093/bioinformatics/bth315, PMID: 15145805

[B38] Manni M. Berkeley M. R. Seppey M. Simão F. A. Zdobnov E. M. (2021). BUSCO update: novel and streamlined workflows along with broader and deeper phylogenetic coverage for scoring of eukaryotic, prokaryotic, and viral genomes. Mol. Biol. Evol. 38, 4647–4654. doi: 10.1093/molbev/msab199, PMID: 34320186 PMC8476166

[B39] Marçais G. Kingsford C. (2011). A fast, lock-free approach for efficient parallel counting of occurrences of k-mers. Bioinformatics 27, 764–770. doi: 10.1093/bioinformatics/btr011, PMID: 21217122 PMC3051319

[B40] Mirarab S. Reaz R. Bayzid M. S. Zimmermann T. Swenson M. S. Warnow T. (2014). ASTRAL: genome-scale coalescent-based species tree estimation. Bioinformatics 30, i541–i548. doi: 10.1093/bioinformatics/btu462, PMID: 25161245 PMC4147915

[B41] Nguyen L. T. Schmidt H. A. Von Haeseler A. Minh B. Q. (2015). IQ-TREE: a fast and effective stochastic algorithm for estimating maximum-likelihood phylogenies. Mol. Biol. Evol. 32, 268–274. doi: 10.1093/molbev/msu300, PMID: 25371430 PMC4271533

[B42] Niu S. Li J. Bo W. Yang W. Zuccolo A. Giacomello S. . (2022). The Chinese pine genome and methylome unveil key features of conifer evolution. Cell 185, 1–14. doi: 10.1016/j.cell.2021.12.006, PMID: 34965378

[B43] Ouadi S. Sierro N. Goepfert S. Bovet L. Glauser G. Vallat A. . (2022). The clove (*Syzygium aromaticum*) genome provides insights into the eugenol biosynthesis pathway. Commun. Biol. 5, 684. doi: 10.1038/s42003-022-03618-z, PMID: 35810198 PMC9271057

[B44] Parra G. Blanco E. Guigó R. (2000). Geneid in drosophila. Genome Res. 10, 511–515. doi: 10.1101/gr.10.4.511, PMID: 10779490 PMC310871

[B45] Parra G. Bradnam K. Korf I. (2007). CEGMA: a pipeline to accurately annotate core genes in eukaryotic genomes. Bioinformatics 23, 1061–1067. doi: 10.1093/bioinformatics/btm071, PMID: 17332020

[B46] Peng Z. Song L. Chen M. Liu Z. Yuan Z. Wen H. . (2024). Neofunctionalization of an OMT cluster dominates polymethoxyflavone biosynthesis associated with the domestication of citrus. Proc. Natl. Acad. Sci. 121, e2321615121. doi: 10.1073/pnas.2321615121, PMID: 38530892 PMC10998556

[B47] Potter S. C. Luciani A. Eddy S. R. Park Y. Lopez R. Finn R. D. (2018). HMMER web server: 2018 update. Nucleic Acids Res. 46, W200–W204. doi: 10.1093/nar/gky448, PMID: 29905871 PMC6030962

[B48] Price A. L. Jones N. C. Pevzner P. A. (2005). *De novo* identification of repeat families in large genomes. Bioinformatics 21, i351–i358. doi: 10.1093/bioinformatics/bti1018, PMID: 15961478

[B49] Reis M. Yang Z. (2011). Approximate likelihood calculation on a phylogeny for Bayesian estimation of divergence times. Mol. Biol. Evol. 28, 2161–2172. doi: 10.1093/molbev/msr045, PMID: 21310946

[B50] Robe K. Izquierdo E. Vignols F. Rouached H. Dubos C. (2021). The coumarins: secondary metabolites playing a primary role in plant nutrition and health. Trends Plant Sci. 26, 248–259. doi: 10.1016/j.tplants.2020.10.008, PMID: 33246890

[B51] Sharifi-Rad J. Cruz-Martins N. López-Jornet P. Lopez E. P. F. Harun N. Yeskaliyeva B. . (2021). Natural coumarins: exploring the pharmacological complexity and underlying molecular mechanisms. Oxid. Med. Cell. Longevity 2021, 6492346. doi: 10.1155/2021/6492346, PMID: 34531939 PMC8440074

[B52] Simão F. A. Waterhouse R. M. Ioannidis P. Kriventseva E. V. Zdobnov E. M. (2015). BUSCO: assessing genome assembly and annotation completeness with single-copy orthologs. Bioinformatics 31, 3210–3212. doi: 10.1093/bioinformatics/btv351, PMID: 26059717

[B53] Stamatakis A. (2014). RAxML version 8: a tool for phylogenetic analysis and post-analysis of large phylogenies. Bioinformatics 30, 1312–1313. doi: 10.1093/bioinformatics/btu033, PMID: 24451623 PMC3998144

[B54] Stanke M. Schöffmann O. Morgenstern B. Waack S. (2006). Gene prediction in eukaryotes with a generalized hidden Markov model that uses hints from external sources. BMC. Bioinformatics 7, 62. doi: 10.1186/1471-2105-7-62, PMID: 16469098 PMC1409804

[B55] Taylor D. W. Hickey L. J. (1992). Phylogenetic evidence for the herbaceous origin of angiosperms. Plant System Evol. 180, 137–156. doi: 10.1007/BF00941148

[B56] Trapnell C. Williams B. A. Pertea G. Mortazavi A. Kwan G. Van Baren M. J. . (2010). Transcript assembly and quantification by RNA-Seq reveals unannotated transcripts and isoform switching during cell differentiation. Nat. Biotechnol. 28, 511–515. doi: 10.1038/nbt.1621, PMID: 20436464 PMC3146043

[B57] Wang P. Fan Z. Wei W. Yang C. Wang Y. Shen X. . (2023). Biosynthesis of the plant coumarin osthole by engineered *Saccharomyces cerevisiae*. ACS Synthetic Biol. 12, 2455–2462. doi: 10.1021/acssynbio.3c00321, PMID: 37450901

[B58] Wang Y. Tang H. DeBarry J. D. Tan X. Li J. Wang X. . (2012). MCScanX: a toolkit for detection and evolutionary analysis of gene synteny and collinearity. Nucleic Acids Res. 40, e49. doi: 10.1093/nar/gkr1293, PMID: 22217600 PMC3326336

[B59] Wendel J. F. Jackson S. A. Meyers B. C. Wing R. A. (2016). Evolution of plant genome architecture. Genome Biol. 17, 37. doi: 10.1186/s13059-016-0908-1, PMID: 26926526 PMC4772531

[B60] Xu Z. Wang H. (2007). LTR_FINDER: an efficient tool for the prediction of full-length LTR retrotransposons. Nucleic Acids Res. 35, W265–W268. doi: 10.1093/nar/gkm286, PMID: 17485477 PMC1933203

[B61] Yang Z. (2007). PAML 4: phylogenetic analysis by maximum likelihood. Mol. Biol. Evol. 24, 1586–1591. doi: 10.1093/molbev/msm088, PMID: 17483113

[B62] Zhang M. Liu D. Fan G. Wang R. Lu X. Gu Y. . (2016). Constituents from Chloranthaceae plants and their biological activities. Heterocyclic Commun. 22, 175–220. doi: 10.1515/hc-2016-0084

[B63] Zhang X. Zhang S. Zhao Q. Ming R. Tang H. (2019). Assembly of allele-aware, chromosomal-scale autopolyploid genomes based on Hi-C data. Nat. Plants 5, 833–845. doi: 10.1038/s41477-019-0487-8, PMID: 31383970

