# Supplementary material for: Isofraxidin biosynthesis in Chloranthus: genomic insights into metabolic evolution of an early angiosperm phytoalexin
Source: Front Plant Sci. 2026 Jan 14;16:1694195. doi: 10.3389/fpls.2025.1694195 (PMC12847343; doi:10.3389/fpls.2025.1694195)
Supplement: Supplementary file 1 [file DataSheet1.docx]

**Supplementary Figures**

**
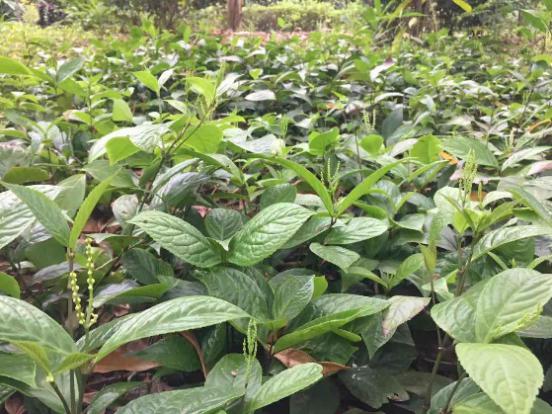

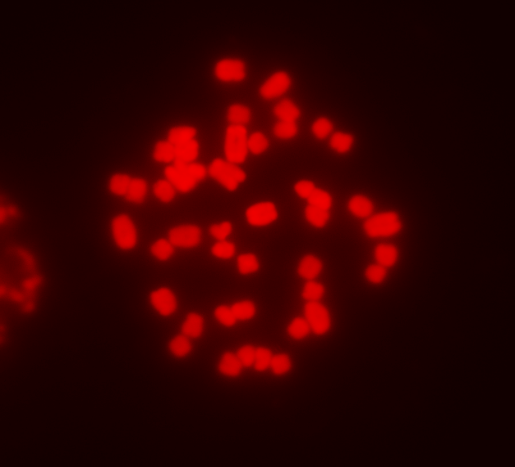
**

**Figure S1.** Flowering morphology and cytogenetic karyotype of *C. spicatus*.


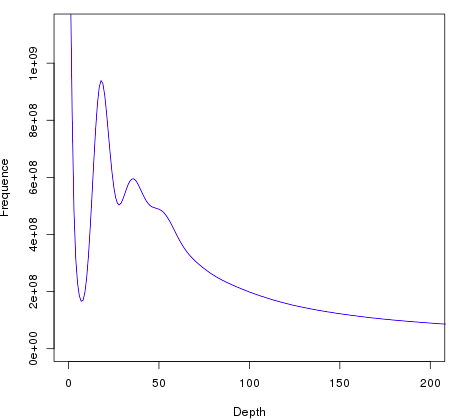


**​Figure S2.** *K*-mer frequency distribution of the *C. spicatus* genome.


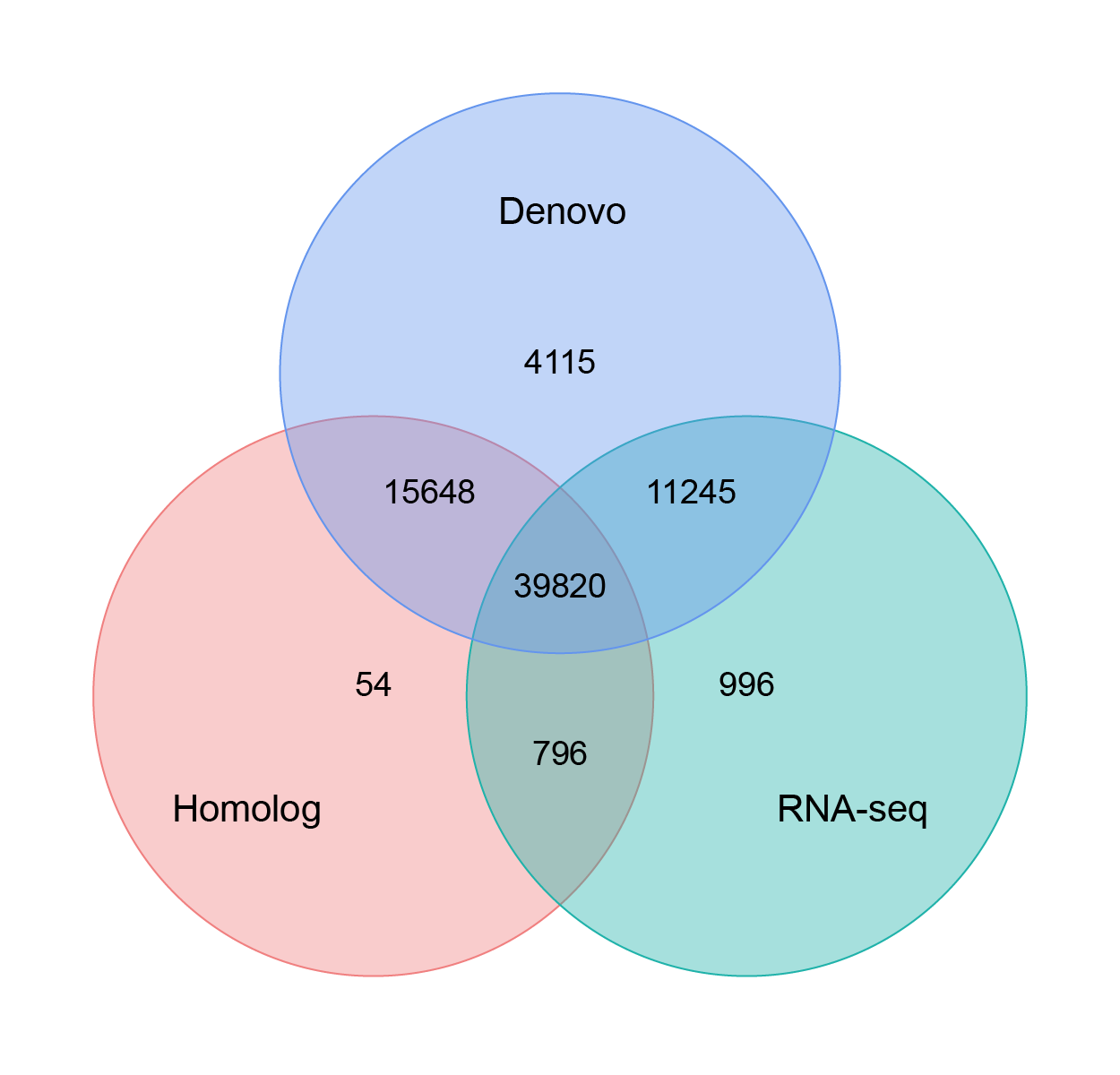


**​Figure S3.** Validation metrics of gene set annotations based on three methodological approaches, with evidence support defined by gene overlap exceeding 50%.


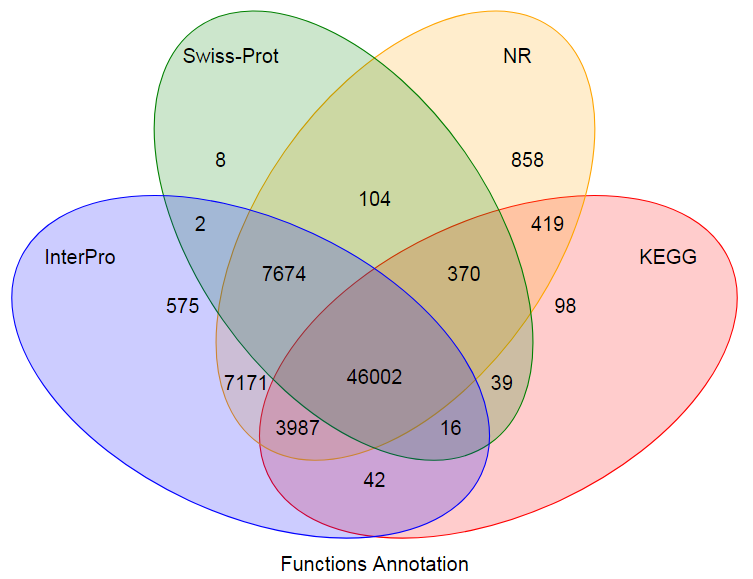


**Figure S4.** Genome-wide gene functional annotation using four integrated databases.


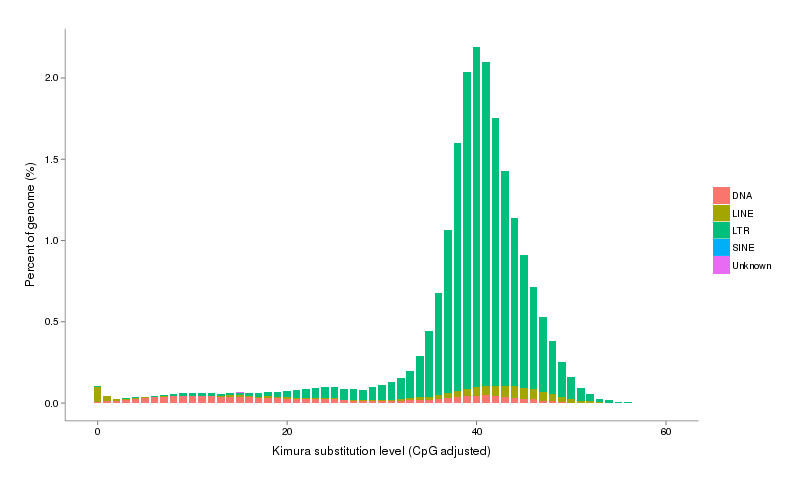


**Figure S5.** Genomic landscape of transposable element (TE) distribution.


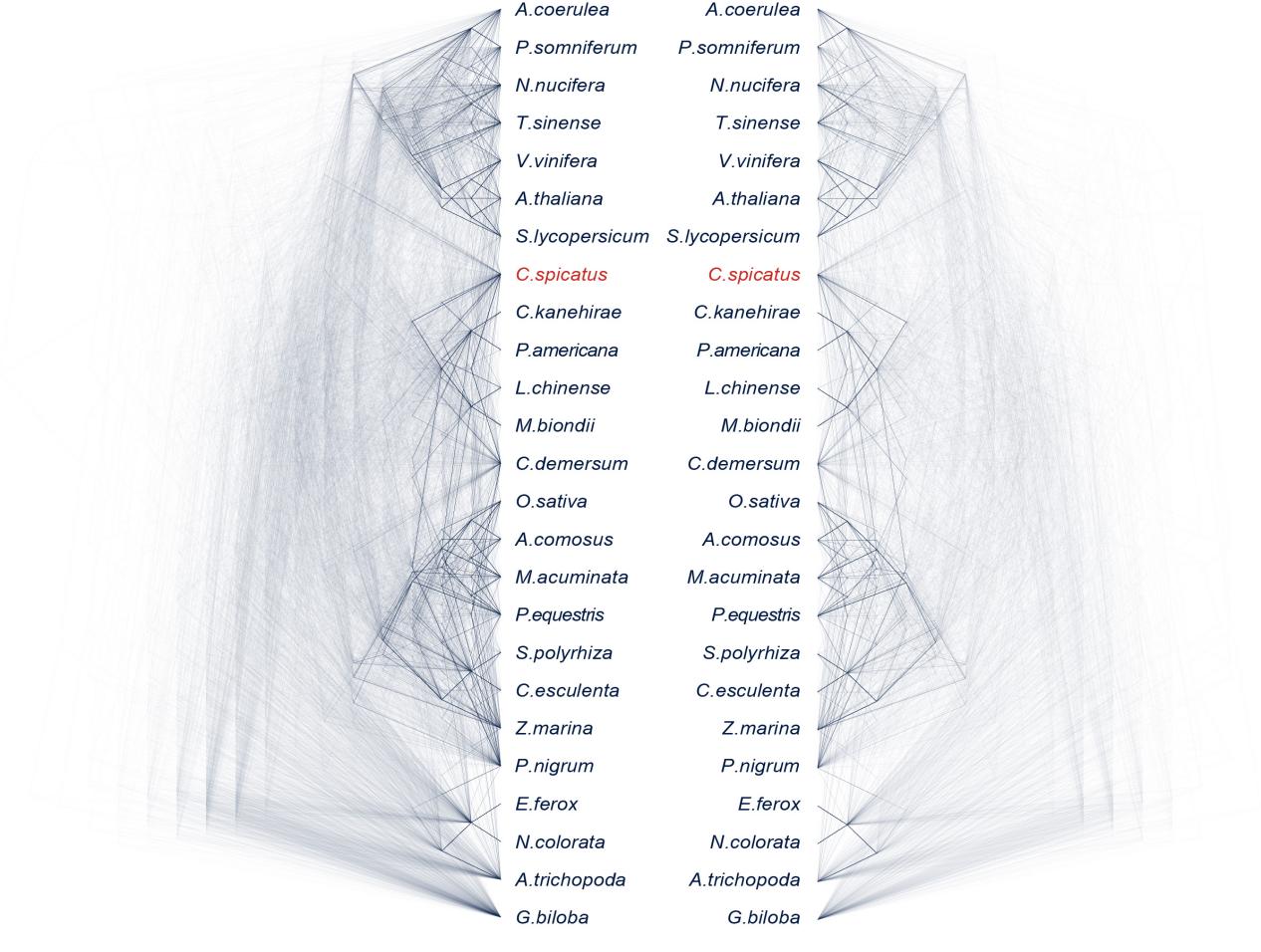


**​​Figure S6.** Phylogenetic density trees generated from two gene sets (n=1092, n=517) via concatenation approach.
